# Contribution of parental and school personnel smoking to health risk behaviours among Finnish adolescents

**DOI:** 10.1186/1471-2458-9-382

**Published:** 2009-10-09

**Authors:** Marianna Virtanen, Minna Pietikäinen, Mika Kivimäki, Pauliina Luopa, Jukka Jokela, Marko Elovainio, Jussi Vahtera

**Affiliations:** 1 Finnish Institute of Occupational Health, Topeliuksenkatu 41 a A, FIN-00250, Helsinki, Finland; 2 National Institute for Health and Welfare, P.O. Box 220, 00531 Helsinki, Finland; 3 University College London, Department of Epidemiology and Public Health, 1-19 Torrington Place, London WC1E 6BT, UK; 4 University of Jyväskylä, Faculty of Sport and Health Sciences, P.O. Box 35, 40014 University of Jyväskylä, Finland; 5 University of Turku, Department of Public Health and Turku University Hospital, Turku, Finland

## Abstract

**Background:**

This study compared parental smoking with school personnel smoking in relation to adolescents' smoking behaviours, alcohol use, and illicit drug use.

**Methods:**

A cross-sectional survey for 24,379 adolescents was linked to a survey for 1946 school employees in 136 Finnish schools in 2004-2005. Surveys included smoking prevalence reported by school staff, adolescents' reports of school staff and parental smoking, adolescents' own smoking behaviours, alcohol use, and illicit drug use. Multilevel analyses were adjusted for individual and school-level confounding factors.

**Results:**

Parental smoking was associated with all health risk behaviours among both sexes (risk range 1.39 to 1.95 for other outcomes; Odds Ratio OR for smoking cessation 0.64, 95% Confidence Interval CI: 0.57, 0.72 among boys, 0.72; 0.64, 0.81 among girls). Among boys, high vs. low smoking prevalence among school personnel was associated with higher probability of smoking (OR 1.19; 95% CI 1.01,1.41), higher frequency of smoking during school time (Cumulative Odds Ratio COR 1.81; 95% CI 1.32, 2.48), frequent alcohol use (OR 1.23; 95% CI 1.01, 1.50), illicit drug use (OR 1.40; 95% CI 1.16, 1.69), and higher odds of reporting adults smoking at school (COR 1.51; 95% CI 1.09, 2.09). Among girls, high smoking prevalence among school personnel was related to higher odds of smoking (OR 1.18; 95% CI 1.02, 1.37) and lower odds of smoking cessation (OR 0.84; 95% CI 0.72, 0.99).

**Conclusion:**

Parental smoking and school personnel smoking are both associated with adolescents' health risk behaviours but the association of parental smoking seems to be stronger.

## Background

Unhealthy lifestyle among adolescents is a globally growing concern and there is an increasing awareness that social environment has an effect on adolescent health behaviours [1,2]. Among the social influences that affect adolescent health behaviours are the behaviour of friends, family members and other role models [3-8], and school culture [1,2,9-11]. There is also some, although inconsistent, evidence that school-based interventions and restrictions can reduce health-risk lifestyle among pupils [11-13]. Several studies on school personnel have focussed on smoking behaviour [1,13-18]. No association was found between teachers' and pupils' smoking in three ecological studies [13-15]. Two studies found an association between teacher's smoking and smoking prevalence among boys but not among girls [16,17]. A further study applied multilevel analysis [18] and reported a mixed pattern of non-significant associations between teachers' and pupils' smoking behaviours. Between-school variance in alcohol and cannabis use has also been reported [19,20].

In this study, we combined responses from two independent surveys: a classroom survey for pupils, and a personnel survey for teachers and other staff in 136 schools in Finland. We examined the contribution of two significant adult groups affecting adolescent development; i.e. the extent to which parental smoking and school personnel smoking are associated with adolescents' health risk behaviours. Although comparisons between peer and parental associations have been made in earlier research [21,22], we are not aware of any studies about comparisons made between parents and school personnel. We additionally examined the relationship between school personnel smoking and adolescents' smoking during school time and their perceptions of staff smoking behaviour at school. According to Finnish legislation, smoking is forbidden in schools and school areas.

## Methods

### Study design

Data were obtained from two independent studies: the Finnish school health promotion study [23,24] focusing on adolescent health and health behaviours and the 10-Town study focusing on health of local government personnel, including schools [25,26]. The nationwide Finnish school health promotion study is a classroom survey that has been implemented since 1995. The study covers virtually all 8th and 9th grades of secondary schools (aged 14-16 years) and the first and second grades of upper secondary schools (aged 16-18 years). The survey has been approved by the ethics committee of Tampere University Hospital. The students responded to the survey on their health and lifestyle habits either in May 2004 or in May 2005 (N = 25 879, response rate 84%).

In the 10-Town study, local government personnel of the towns participating responded to a questionnaire between October 2004 and January 2005 (response rate 65%; school personnel response rate 54%). The study was approved by the ethics committee of the Finnish Institute of Occupational Health. A total of 150 (83%) secondary schools participated both in the Finnish school health promotion study and the 10-Town study. Of them, five schools were excluded because they had less than the required minimum of four respondents from personnel. Nine more schools were excluded because the number of pupil respondents was less than 30, resulting in a sample of 136 schools. The total number of school personnel respondents was 1946 (495 men, 1451 women), the mean number of respondents per school being 14 (range 4 to 45, SD = 6.9). Of the school personnel respondents, 1856 (95%) were teachers (84 head teachers). The remaining 5% were mainly administrative staff and canteen workers. We obtained information on average smoking prevalence among teachers and other personnel in each school from the survey responses and linked this data to the data on pupils.

In the 136 schools, the number of pupil respondents was 25,879, out of whom 24,379 (94%) had provided complete data on demographics and parental smoking. The mean number of pupil respondents per school was 190 (range 32 to 531, SD = 76.2).

### Measures

Data on parental smoking and adolescent's health behaviours and perceptions were derived from the Finnish School Health Promotion Study. Smoking frequency was assessed with two questions. First, pupils were asked: "How many cigarettes, pipes, or cigars have you smoked this far?" Respondents who answered "None" were categorized as non-smokers. Current smoking status was enquired as follows: "Which of the following alternatives describes your current smoking?" with response alternatives "None; less than once a week; at least once a week but not daily; every day; have quit smoking". Those who responded 'none' or had quit smoking were categorized as non-smokers, and all other respondents were categorised as smokers. Further, a dichotomous variable for smoking cessation was derived: 1 = current smoker; 2 = has quit smoking.

Smoking intensity during school time was calculated for those who reported regular smoking (at least once a week) with the following two questions: "How often do you smoke at school or in the school area?" and "How often do you smoke in the vicinity of your school during school time?" with response alternatives 1 = never; 2 = sometimes; 3 = every day. If the adolescent answered "never" to both questions, we coded the answer as "1 = never" and if the answer was "every day" to either of the two questions we coded the answer as "3 = every day". All other combinations were registered as "2 = sometimes". Adolescents' reports of school personnel smoking was assessed by a single question about whether teachers and other school personnel smoked at school or in the school area (1 = yes, every day; 2 = yes, sometimes; 3 = no; 4 = can not say). Those who chose the last alternative were omitted and the scale was reversed.

Alcohol use frequency was asked with the following question: "How often do you use alcohol, for example half a bottle of beer or more?" with response alternatives 1 = I don't use alcohol; 2 = less than once a month; 3 = about once a month; 4 = about twice a month; 5 = at least once a week. A dichotomous variable for weekly alcohol use was formulated but additional analyses were done treating alcohol use frequency as a continuous variable. The question assessing binge drinking was: "How often do you get really drunk?" with response alternatives: 1 = Once a week or more often; 2 = about 1-2 times a month; 3 = rarely than that; 4 = never. A dichotomous variable was formulated combining alternatives 1 and 2 (cases), and 3 and 4 (non-cases). One-month illicit drug use was asked with the following question: "Think back to the past 30 days. How many times during that time have you used drugs with the intention to become intoxicated (for example, thinner, glue, pharmaceuticals, alcohol and pharmaceuticals together, cannabis, ecstasy, subutex, heroin, cocaine, amphetamine, or LSD?)" with response alternatives 1 = not at all; 2 = once; 3 = 2-4 times; 4 = 5 times or more. From these, a dichotomous variable was formed as follows: 1 = no; 2 = at least once.

The adolescents' sex and grade level were derived from the survey responses. Each pupil's socioeconomic background was ascertained from two questions asking separately about the mother's and father's education: 'What is the highest education your parents have performed?' (1 = comprehensive school/comprehensive school and vocational school; 2 = upper secondary school/upper secondary school and institute; 3 = college or university). If both the mother's and father's education were reported, the highest education level between them was chosen.

School-level percentage of pupils with low parental education was derived from the measure of individual responses about the parental education. Pupils with low parental education were those whose parents' education level was vocational school or less (range between schools varied from 4% to 68%). Three groups of equal size were then formed: 1 = <22%; 2 = 22-32%; 3 = 33% or more of pupils.

Smoking prevalence among school personnel was obtained from the questionnaire in the 10-Town study asking "Do you smoke or have you previously smoked regularly, that is, daily or nearly daily?", and "If you have smoked, do you still smoke regularly?" Of these, a dichotomous variable was calculated (1 = non-smoker; 2 = smoker). Percentage of smokers at work-unit level was aggregated using work-unit codes derived from the employers' registers. This measure was divided into three categories of about equal size: 1 = 0-4%; 2 = >4-13% and 3 = >13% smokers. Using information on each respondent's work-unit code, those data were linked to the data of each respondent of the Finnish school health promotion study.

### Data analysis

We used multilevel analyses in the SAS 9.1 GLIMMIX procedure to assess the school-level and individual-level associations. For dichotomous outcome variables, we used multilevel random intercept models in binary logistic regression models and calculated odds ratios (OR) and their 95 percent confidence intervals (CI). For outcome variables with more than two categories, we used multilevel random intercept models with multinomial logistic regression procedure calculating cumulative odds ratios (COR) and their 95% CIs. We made adjustments for pupils' grade level, parent's socioeconomic position, the proportion of pupils at school with parents with low education, and the proportion of non-teacher respondents among school personnel. Analyses were made separately for boys and girls.

## Results

We found significant between-school variance in all measured outcome variables (all *p*-values < 0.01) for both boys and girls. The individual and school-level characteristics for boys and girls are shown in Table 1. Significant differences between sexes were found in almost all variables: girls dominated 1st and 2nd grades of high school, they had more often parents with lower education, were more likely to smoke (32% of the girls vs 28% of the boys smoked), have a smoking parent (38% vs 36%), and had stopped smoking less often than boys (33% vs 40%). However, girls were less likely to smoke during school time or report seeing school personnel smoking. Girls also used less often alcohol and got really drunk less often but had used illicit drugs slightly more often than boys (10% vs 9%). Boys were slightly more often in schools with a high proportion of pupils of a manual socioeconomic background. The proportion of smokers among school personnel (Mean 8.8%, S.D. 9.1) was not significantly associated with pupil sex distribution.

**Table 1:** Individual, family and school level characteristics among boys and girls

	**Boys (n = 11,609)**	**Girls (n = 12,770)**
**Individual and family characteristics**	**n (%)**	**n (%)**
Grade level		
8th (secondary school)	3967 (34)	3892 (30)
9th (secondary school)	3845 (33)	3910 (31)
1st (upper secondary school)	2079 (18)	2704 (21)
2nd (upper secondary school)	1718 (15)	2264 (18)
Socioeconomic background (parental education)		
Comprehensive school	637 (5)	828 (6)
Comprehensive school and vocational school	2176 (19)	3052 (24)
Upper secondary school/upper secondary school + institute	3095 (27)	3359 (26)
College or university	5701 (49)	5531 (43)
Parental smoking		
No	7391 (64)	7930 (62)
Yes	4218 (36)	4840 (38)
Own current smoking		
No	8219 (72)	8559 (68)
Yes	3213 (28)	4005 (32)
Smoking cessation (among those who had ever smoked)		
No	3213 (60)	4005 (67)
Yes	2165 (40)	1940 (33)
Smoking during school time (among regular smokers)		
Never	269 (12)	381 (15)
Sometimes	569 (26)	732 (28)
Every day	1356 (62)	1457 (57)
Reporting personnel smoking at school		
Never	2514 (35)	2822 (39)
Sometimes	2196 (30)	2281 (31)
Every day	2562 (35)	2179 (30)
Alcohol use frequency		
Less than weekly	9831 (85)	11,390 (89)
At least once a week	1736 (15)	1343 (11)
Binge drinking (gets really drunk)		
Never or rarely than once a month	8526 (74)	10,130 (80)
At least once a month	3034 (26)	2605 (20)
Illicit drug use during the past month		
No	10,518 (91)	11,482 (90)
Yes	1042 (9)	1245 (10)
School-level characteristics		
Proportion (%) of pupils at school with low parental education*		
< 22	3739 (32)	4273 (33)
≥22 < 33	3847 (33)	4318 (34)
≥33	4023 (35)	4179 (33)
Proportion (%) of smokers among school personnel		
≤4	3915 (34)	4432 (35)
> 4 ≤ 13	4036 (35)	4469 (35)
> 13	3658 (32)	3869 (30)

*Parental education level comprehensive school or comprehensive school and vocational school.

Note. All differences between boys and girls were statistically significant except the difference in the prevalence of school personnel smoking (*p *= 0.095).

Table 2 presents the fully adjusted multivariate multilevel models of the association between individual-level and school-level covariates and outcome measures. In the fully adjusted models, female sex, higher grade level, lower parental education, and attending a school with a higher proportion of pupils with low level of parental education were related to pupil's smoking. Among those who had smoked at least sometimes, girls and those with lower parental education had lower odds to have quit smoking. Girls had also lower odds of smoking during school time whereas pupils on higher grade level and those with lower parental education as well as those attending a school with a higher proportion of pupils with low-education parents had higher odds of frequent smoking during school time. The odds ratio of reporting school personnel smoking was higher among boys than girls. In addition, pupils with low-education parents and those from schools with a high proportion of pupils with low-education parents had higher odds of reporting school personnel smoking. The odds ratios for using alcohol at least weekly and getting really drunk at least once a month were higher among boys, higher-grade pupils and those with low parental education. Pupil socioeconomic composition at school was not related to individual alcohol use. The odds of reporting illicit drug use was slightly higher among girls than boys. Lower parental education and higher proportion of pupils at school having parents with low education were associated with greater odds for drug use.

**Table 2:** Association of individual and school level characteristics with adolescents' health risk behaviours and their perceptions of school personnel smoking.

	**Smoking prevalence**	Smoking cessation*	**Smoking intensity during school time†**	**Reporting personnel smoking at school**	**Weekly alcohol use**	**Binge drinking**	**One-month drug use**
	
	**OR (95% CI)‡**	**OR (95% CI)‡**	**COR (95% CI)‡**	**COR (95% CI)‡**	**OR (95% CI)‡**	**OR (95% CI)‡**	**OR (95% CI)‡**
	
Sex							
Boy	1.00	1.00	1.00	1.00	1.00	1.00	1.00
Girl	1.17 (1.11-1.24)	0.72 (0.66-0.78)	0.73 (0.65-0.83)	0.78 (0.73-0.83)	0.63 (0.58-0.68)	0.70 (0.65-0.74)	1.10 (1.00-1.20)
Grade level							
8th (secondary school)	1.00	1.00	1.00	1.00	1.00	1.00	1.00
9th (secondary school)	1.50 (1.40-1.62)	0.86 (0.78-0.96)	1.30 (1.12-1.50)	1.05 (0.97-1.13)	1.77 (1.59-1.98)	1.59 (1.47-1.73)	1.17 (1.05-1.30)
1st (upper secondary school)	1.42 (1.24-1.63)	0.93 (0.81-1.08)	1.69 (1.26-2.28)	0.78 (0.55-1.12)	1.95 (1.63-2.33)	1.79 (1.55-2.07)	0.88 (0.75-1.04)
2nd (upper secondary school)	1.98 (1.72-2.27)	0.88 (0.77-1.02)	1.98 (1.47-2.66)	1.01 (0.70-1.44)	4.24 (3.57-5.04)	3.11 (2.69-3.59)	0.98 (0.83-1.16)
Parental education							
I highest	1.00	1.00	1.00	1.00	1.00	1.00	1.00
II	1.02 (0.95-1.09)	0.99 (0.90-1.09)	1.06 (0.91-1.23)	0.97 (0.89-1.04)	1.00 (0.90-1.10)	1.05 (0.97-1.13)	1.03 (0.93-1.15)
III	1.28 (1.19-1.38)	0.87 (0.79-0.97)	1.36 (1.16-1.59)	1.04 (0.96-1.14)	1.21 (1.09-1.34)	1.32 (1.21-1.43)	1.14 (1.01-1.27)
IV lowest	1.56 (1.39-1.75)	0.72 (0.61-0.84)	1.42 (1.13-1.77)	1.15 (1.00-1.32)	1.60 (1.37-1.87)	1.55 (1.37-1.76)	1.48 (1.25-1.75)
Proportion (%) of pupils at school with low parental education§							
< 22	1.00	1.00	1.00	1.00	1.00	1.00	1.00
≥ 22 < 33	1.11 (0.96-1.28)	0.87 (0.76-1.00)	1.16 (0.85-1.59)	1.25 (0.83-1.88)	0.90 (0.75-1.08)	1.04 (0.89-1.21)	1.26 (1.08-1.47)
≥ 33	1.21 (1.05-1.41)	0.88 (0.77-1.02)	1.57 (1.15-2.14)	1.53 (1.02-2.29)	0.99 (0.88-1.19)	1.11 (0.96-1.30)	1.35 (1.15-1.58)

*Among ever smokers (n = 11,323).

† Smokes at school and/or in the vicinity of school during schooldays; among regular smokers (n = 4764).

‡ Fully adjusted model.

§ Parental education level comprehensive school or comprehensive school and vocational school.

Note. Multilevel analysis, all variables entered simultaneously. OR, odds ratio; COR, cumulative odds ratio; CI, confidence interval.

Table 3 shows fully adjusted multilevel models of the association of parental smoking and school personnel smoking with pupil outcomes, separately for boys and girls. Among both sexes, parental smoking was strongly and consistently associated with all health risk behaviours with elevated risks ranging between 1.52 and 1.95 among boys and 1.39 and 1.93 among girls. Parental smoking was related with 0.64 times lower odds of smoking cessation among boys and 0.72 times lower odds of cessation among girls who had smoked at least sometimes. Odds ratio for smoking was 1.19 and 1.18 for boys and girls in schools with a high proportion of smokers among school personnel compared to those in schools with a low proportion of smokers among school personnel. The corresponding odds ratio for smoking cessation in girls was 0.84. Among boys, a high percentage of smokers in school staff was associated with a cumulative odds ratio of 1.81 for smoking during schooldays, a cumulative odds ratio of 1.51 for reporting school personnel smoking at school, odds ratio of 1.23 for weekly alcohol use, and an odds ratio of 1.40 for drug use. When alcohol use was analysed using multinomial logistic regression, cumulative odds ratio for frequent alcohol use in schools with high prevalence of staff smokers was 1.14 (95% CI 0.99-1.31) among boys and 1.07 (95% CI 0.94-1.23) among girls (not shown in the table).

**Table 3:** Association of parental smoking and school personnel smoking with adolescents' health risk behaviours and their perceptions of school personnel smoking.

	**Smoking**	Smoking cessation*	**Smoking during school time†**	**Reporting personnel smoking at school**	**Weekly alcohol use**	**Binge drinking**	**One-month drug use**
	
	**OR (95% CI)‡**	**OR (95% CI)‡**	**COR (95% CI)‡**	**COR (95% CI)‡**	**OR (95% CI)‡**	**OR (95% CI)‡**	**OR (95% CI)‡**
	
Boys							
Parental smoking							
No	1.00	1.00	1.00	1.00	1.00	1.00	1.00
Yes	1.95 (1.78-2.13)	0.64 (0.57-0.72)	1.52 (1.27-1.83)	1.16 (1.06-1.27)	1.55 (1.39-1.73)	1.79 (1.63-1.96)	1.72 (1.50-1.96)
School personnel smoking prevalence							
0 ≤ 4%	1.00	1.00	1.00	1.00	1.00	1.00	1.00
> 4 ≤ 13%	0.92 (0.78-1.10)	1.11 (0.93-1.33)	1.55 (1.12-2.16)	1.14 (0.82-1.59)	0.98 (0.80-1.21)	0.90 (0.76-1.07)	1.25 (1.03-1.52)
> 13%	1.19 (1.01-1.41)	0.90 (0.75-1.07)	1.81 (1.32-2.48)	1.51 (1.09-2.09)	1.23 (1.01-1.50)	1.09 (0.93-1.29)	1.40 (1.16-1.69)
Girls							
Parental smoking							
No	1.00	1.00	1.00	1.00	1.00	1.00	1.00
Yes	1.93 (1.78-2.10)	0.72 (0.64-0.81)	1.39 (1.18-1.65)	1.02 (0.93-1.12)	1.82 (1.62-2.06)	1.79 (1.63-1.96)	1.75 (1.55-1.97)
School personnel smoking prevalence							
0 ≤ 4%	1.00	1.00	1.00	1.00	1.00	1.00	1.00
> 4 ≤ 13%	1.01 (0.86-1.17)	0.84 (0.71-0.98)	1.26 (0.89-1.78)	1.12 (0.78-1.63)	0.92 (0.75-1.13)	1.00 (0.83-1.19)	1.09 (0.92-1.30)
> 13%	1.18 (1.02-1.37)	0.84 (0.72-0.99)	1.23 (0.88-1.72)	1.39 (0.97-2.00)	1.14 (0.94-1.39)	1.11 (0.93-1.33)	1.14 (0.96-1.35)

*Among ever smokers (n = 11,323)

† Smokes at school and/or nearby school during schooldays; among regular smokers (n = 4764)

‡ Multilevel analysis adjusted for student's grade level, parental education, school personnel smoking/parental smoking, the proportion of pupils with low parental education at school, and the proportion of non-teacher respondents at school

Note. OR, odds ratio; COR, cumulative odds ratio; CI, confidence interval

It is possible that boys more frequently report school personnel smoking because they themselves smoke more often during school time for example, on the school backyards. Therefore we ran a sensitivity analysis on non-smokers only. We found that among the non-smokers, the cumulative odds ratio of reporting that personnel smoke at school were 1.16 (95% CI 1.08 to 1.26) for boys when compared with girls. Among boys, an association was also found between a higher percentage of school staff smokers and higher odds of boys seeing school personnel smoke (cumulative odds ratio 1.49, 95% CI 1.05 to 2.11).

## Discussion

This study examined the contribution of smoking of two significant adult groups - parents and school personnel - to various health risk behaviours and perceptions among adolescents. We found that parental smoking was strongly and consistently associated with all health risk behaviours studied among both sexes. Furthermore, a high percentage of smoking among school personnel was related to smoking both among boys and girls and lower odds of smoking cessation among girls. Furthermore, among boys, higher proportion of staff smokers at school was related to higher odds of smoking at school, reporting staff smoking at school, higher use of alcohol, and higher use of illicit drugs. Although parental smoking and school personnel smoking were both associated with adolescents' health risk behaviours, the association of parental smoking was stronger.

We found that smoking and having a smoking parent was related to female sex. Girls had also stopped smoking less often than boys. However, boys smoked more often during school time, used more alcohol and reported more often binge drinking than girls. One-month drug use was slightly more common among girls. The prevalences found in our study are not easy to compare with other studies due to different methodology and age range used, especially with regard to alcohol use and illicit drug use. We found that 28% of boys and 32% of girls were smokers. However, adolescents who smoked less than once a week were included as smokers. Prevalence of smoking has varied between 3% and 30% in other studies [27-31], and in line with our results, higher prevalence of smoking among girls than among boys has been reported but smoking intensity has been greater among smoking boys [29]. Girls' slightly higher prevalence of smoking may partially be explained by higher use of non-smoke tobacco, snuff, among boys [27]. Binge drinking seems to be more common among boys than girls. In the U.S. 11% of the boys and 10% of the girls aged 12-17 years had drank five or more drinks of alcohol at least once during the past month [28] which is less than in our study (26% of the boys and 20% of the girls said they binge at least monthly). However, of 15-16-year-old adolescents in France and U.K., 16% and 33% of the boys and 7% and 27% of the girls had binged at least three times in the past month [31]. Nine percent of the boys and 10% of the girls reported one-month illicit drug use (cannabis or other) in our study which implicates practically no sex differences in one hand, and less common drug use in Finland than in many other countries, on the other hand. In a study of youth aged 15-16 years in France and in the U.K.[31], 25% of the French boys and 19% of the British boys had used cannabis in the past month. Corresponding figures for the girls were 19% and 15%. In a cross-national survey of cannabis use among 15-year-olds [32], the highest prevalence of frequent (more than 40 times in life-time) cannabis use was seen in boys in Canada and Switzerland (14%), and in girls in U.S. (6%) and Switzerland (5%). Corresponding figures for the Finnish boys and girls were 1% and 0.2%. In our study, illicit drug use included use of pharmaceuticals which may be more common among girls and in part explain the small sex difference found in drug use. However, Ter Bogt and others argued that the wealthier the country, the smaller are sex differences in substance use [32] although there are probably many other contributing sociocultural and family characteristics predicting adolescent substance use [31].

The proportion of smokers among school personnel was on an average of 8.8% in our study. This kind of relatively low average prevalence of teacher smoking has also been reported in an earlier study [15]. In our study, 65% of the boys and 61% of the girls reported school personnel smoking at school. In a Danish study, 86% of the boys and 88% of the girls said they were exposed to teachers' smoking [33]. Corresponding with our results, that study showed an association between adolescents' reports of teacher smoking at school and their own smoking.

The findings that parental smoking is associated with smoking, alcohol use and illicit drug use are consistent with earlier studies [3-7]. Also in line with earlier research, we found that parental smoking was associated with decreased odds of quitting among adolescents who had previously smoked [34-36]. However, we are not aware of earlier studies showing that among regular smokers, parental smoking is also related to smoking during school time, which may be a proxy measure for smoking intensity.

Earlier studies have mainly focused on smoking and have shown that school context in itself is related to the smoking of pupils [1,2]. Of the possible explanatory factors for school-level variance, pupil's socioeconomic composition has been one of the most important candidates [1]. In our study, the proportion of pupils in school with parents with low education level had an independent association with adolescent's smoking, smoking cessation, smoking during school time, and illicit drug use.

Substance use control policy at school is another important factor which may explain why school affects adolescent smoking and other health risk behaviours [1,37]. In Finland, smoking is forbidden in schools and school areas. Municipalities are responsible for the supervision of the smoking ban, which means that school personnel should take charge in keeping schools smoke-free. However, teachers' perceptions of the school smoking policy as too strict have been associated with smoking among pupils [16]. School staff attitudes towards smoking may be less strict if they themselves smoke. We found that in schools where the proportion of staff smokers is higher, pupils actually see personnel smoking at school. This might be interpreted by the pupils as a "green light" for smoking.

We found that the association between staff smoking and pupil's smoking, alcohol and drug use was stronger for boys than for girls. If staff smoking prevalence is considered as an indicator of role modelling or attitudes towards health risk behaviours, girls may be only be affected when making the decision whether to smoke or not. In boys, we found an association also with alcohol and illicit drug use. Our finding that boys also report seeing personnel smoke at school more often than girls and that their perception is related to the actual number of staff smokers at school gives support to the hypothesized role model effect [1,8]. There is some evidence that the psychosocial determinants of smoking are somewhat different in boys and girls [38]. Smoking has been hypothesized to represent independence and rebelliousness among girls but a mechanism to cope with social insecurity among boys. Girls have even reported "infuriating parents" as one of the benefits in smoking [39]. That is why girls may also smoke in schools where personnel do not smoke.

However, we found stronger associations of adolescent health risk behaviours with parental smoking than with school personnel smoking. There are at least two possible explanations for this finding. First, the associations found between parents and children may be due to shared genetic factors [40,41]. However, a recent study showed that teenagers are as affected by smoking by a non-biological parent as smoking by their biological parents [42]. We found that parental smoking was associated not only with smoking but also with all forms of health risk behaviours among adolescents. Parental smoking may be a proxy for parenting and supervision style, which in turn may increase risk-taking behaviours among children [6,31].

The methodological advances of our study were the large number of schools and their high participation rate; vast majority of secondary schools and upper secondary schools of the ten towns participated in the study. In Finland, about 98% of comprehensive school education is public and organized by the municipalities, rendering the study widely generalizable. We adjusted the most likely confounders in our analyses, i.e. pupil's grade level, parent's education, parental/school personnel smoking, and the pupils' socioeconomic composition at school. However, there may still be some unmeasured factors which may confound the association between school personnel smoking and health behaviours among pupils. Furthermore, we used odds ratios as association estimates. Although they are relative measures, odds ratios should not be interpreted as risk ratios. Given the relatively high prevalence of our outcome measures (range from 9% to 40%), odds ratios tend to provide greater values than estimates of relative risk.

Response rate in the pupils' survey was 84%. The response rate among school personnel was relatively low, 54%. One methodological consideration relates to selective non-response. For example, if substantially more smokers in the schools with high proportion of smokers than those in the schools with low proportion of smokers were non-respondents, our findings between school personnel high smoking prevalence and adolescent health behaviours would have been overestimated. However, we were not able to test such a bias with our data.

## Conclusion

Unhealthy lifestyle among adolescents has serious effects on health later in life [43]. This study suggests that parental smoking and school personnel smoking are both associated with adolescents' health risk behaviours but the association of parental smoking seems to be stronger. However, as our study was cross-sectional, temporal order between the variables cannot be determined. If these associations are causal, promoting a healthy lifestyle among parents and school personnel may have a favourable effect on adolescent health behaviours.

## Declaration of Competing interests

The authors declare that they have no competing interests.

## Authors' contributions

MV, the principal investigator, planned the study design, analysed data and interpreted the results, and drafted the manuscript. MP, PL, and JV contributed to data collection, planning the study, interpreting the results and drafting the manuscript or revising it critically for important intellectual content. JJ, ME, and MK contributed to conception and design, interpretation of data, and drafting the manuscript or revising it critically for important intellectual content. All authors have given final approval of the version to be published.

## Pre-publication history

The pre-publication history for this paper can be accessed here:



## References

[B1] Aveyard P, Markham WA, Cheng KK (2004). A Methodological and substantive review of the evidence that schools cause pupils to smoke. Soc Sci Med.

[B2] Sellström E, Bremberg S (2006). Is there a "school effect" on pupil outcomes? A review of multilevel studies. J Epidemiol Community Health.

[B3] Geckova A, van Dijk JP, van Ittersum-Gritter T, Groothoff JW, Post D (2002). Determinants of adolescents' smoking behaviour: a literature review. Cent Eur J Public Health.

[B4] Resnick MD, Bearman PS, Blum RW, Bauman KE, Harris KM, Jones J, Tabor J, Beuhring T, Sieving RE, Shew M, Ireland M, Bearinger LH, Udry JR (1997). Protecting adolescents from harm. Findings from the national Longitudinal Study on Adolescent Health. JAMA.

[B5] Rachiotis G, Muula AS, Rudatsikira E, Siziua S, Kyrlesi A, Gourgoulianis K, Hadjichristodoulou C (2008). Factors associated with adolescent cigarette smoking in Greece: results from a cross sectional study (GYTS Study). BMC Public Health.

[B6] Hayatbakhsh MR, Hayatbakhsh MR, Alati R, Hutchinson DM, Jamrozik K, Najman JM, Mamun AA, O'Callaghan M, Bor W (2007). Association of maternal smoking and alcohol consumption with young adults' cannabis use: a prospective study. Am J Epidemiol.

[B7] Kandel DB, Hu M-C, Griesker PC, Schaffran C (2007). On the development of nicotine dependence in adolescence. Drug Alcohol Depend.

[B8] Sargent JD, Beach ML, Adachi-Mejia AM, Gibson JJ, Titus-Ernstoff LT, Carusi CP, Swain SD, Heatherton TF, Dalton MA (2005). Exposure to movie smoking: its relation to smoking initiation among US adolescents. Pediatrics.

[B9] Maes L, Lievens J (2003). Can the school make a difference? A multilevel analysis of adolescent risk and health behaviour. Soc Sci Med.

[B10] Johansen A, Rasmussen S, Madsen M (2006). Health behaviour among adolescents in Denmark: influence of school class and individual risk factors. Scand J Public Health.

[B11] Fletcher A, Bonell C, Hargreaves J (2008). School effects on young people's drug use: A systematic review of intervention and observational studies. J Adolescent Health.

[B12] Thomas R, Perera R (2006). School-based programmes for preventing smoking. Cochrane Database Syst Rev.

[B13] Nilsson M, Stenlund H, Bergstrom E, Weinehall L, Janlert U (2006). It takes two: reducing adolescent smoking uptake through sustainable adolescent-adult partnership. J Adolescent Health.

[B14] Johnson MR, Bewley BR, Banks MH, Bland JM, Clyde DV (1985). Schools and smoking: school features and variations in cigarette smoking by children and teachers. Brit J Educ Psychol.

[B15] Clarke V, White V, Hill D, Borland R (1994). School structural and policy variables associated with student smoking. Tob Contr.

[B16] Murray M, Kiryluk S, Swan AV (1984). School characteristics and adolescent smoking. Results from the MRC/Derbyshire Smoking Study 1974-8 and from a follow up in 1981. J Epidemiol Community Health.

[B17] Bewley BR, Johnson MR, Banks MH (1979). Teachers' smoking. J Epidemiol Community Health.

[B18] De Moor C, Cookson K, Edler JP, Young R, Molgaard CA, Widley M (1992). The association between teacher attitudes, behavioural intentions, and smoking and the prevalence of smoking among seventh-grade students. Adolescence.

[B19] Kuntsche E, Jordan MD (2006). Adolescent alcohol and cannabis use in relation to peer and school factors. Results of multilevel analyses. Drug Alcohol Depend.

[B20] Knibbe RA, Joosten J, Choquet M, Derickx M, Morin D, Monshouwer K (2007). Culture as an explanation for substance-related problems: a cross-national study among French and Dutch adolescents. Soc Sci Med.

[B21] Vitaro F, Wanner B, Brendgen M, Gosselin C, Gendreau PL (2004). Differential contribution of parents and friends to smoking trajectories during adolescence. Addict Behav.

[B22] de Vries H, Candel M, Engels R, Mercken L (2006). Challenges to the peer influence paradigm: results for 12-13 year olds from six European countries from the European Smoking Prevention Framework Approach study. Tob Control.

[B23] Hakala P, Rimpelä A, Salminen JJ, Virtanen SM, Rimpelä M (2002). Back, neck, and shoulder pain in Finnish adolescents: national cross sectional study. BMJ.

[B24] Kaltiala-Heino R, Rimpelä M, Marttunen M, Rimpelä A, Rantanen P (1999). Bullying, depression, and suicidal ideation in Finnish adolescents: School survey. BMJ.

[B25] Vahtera J, Kivimäki M, Pentti J, Linna A, Virtanen M, Virtanen P (2004). Organisational downsizing, sickness absence, and mortality: 10-town prospective cohort study. BMJ.

[B26] Poikolainen K, Vahtera J, Virtanen M, Linna A, Kivimäki M (2005). Alcohol and coronary heart disease risk: is there an unknown confounder?. Addiction.

[B27] Hedman L, Bjerg-Bäcklund A, Perzanowski M, Sundberg S, Rönmark E (2007). Factors related to tobacco use among teenagers. Resp Med.

[B28] Fryar C, Merino MC, Hirsch R, Porter K (2009). Smoking, alcohol use, and illicit drug use reported by adolescents aged 12-17 years: United States, 1999-2004. Natl Health Stat Rep.

[B29] Gold DR, Wang X, Wypij D, Speizer FE, Ware JH, Dockery DW (1996). Effects of cigarette smoking on lung function in adolescent boys and girls. N Engl J Med.

[B30] Edvardsson I, Lendahls L, Håkansson A (2009). When do adolescents become smokers? Annual seven-year population-based follow-up of tobacco habits among 2000 Swedish pupils - an open cohort study. Scand J Prim Health Care.

[B31] Ledoux S, Miller P, Choquet M, Plant M (2002). Family structure, parent-child relationships, and alcohol and other drug use among teenagers in France and the United Kingdom. Alcohol Alcoholism.

[B32] ter Bogt T, Schmid H, Gabbhainn SN, Fotiou A, Vollebergh W (2006). Economic and cultural correlates of cannabis use among mid-adolescents in 31 countries. Addiction.

[B33] Poulsen LH, Osler M, Roberts C, Due P, Damsgaard MT, Holstein BE (2002). Exposure to teachers smoking and adolescent smoking behaviour: analysis of cross sectional data from Denmark. Tob Contr.

[B34] van Zundert RM, Ven MO van de, Engels RC, Otten R, Eijnden RJ van den (2007). The role of smoking-cessation-specific parenting in adolescent smoking-specific cognitions and readiness to quit. J Child Psychol Psychiatry.

[B35] den Exter Blokland EA, Hale WW, Meeus W, Engels RC (2007). Parental support and control and early adolescent smoking: a longitudinal study. Subst Use Misuse.

[B36] Marcus SE, Pahl K, Ning Y, Brook JS (2007). Pathways to smoking cessation among African American and Puerto Rican young adults. Am J Public Health.

[B37] Adelman WP, Duggan AK, Hauptman P, Joffe A (2001). Effectiveness of a high school smoking cessation program. Pediatrics.

[B38] Clayton S (1991). Gender differences in psychosocial determinants of smoking. J School Health.

[B39] Urberg K, Robbins RL (1981). Adolescents perceptions of the costs and benefits associated with cigarette smoking: sex differences and peer influence. J Youth Adolesc.

[B40] Kendler KS, Karkowski LM, Corey LA, Prescott CA, Neale MC (1999). Genetic and environmental risk factors in the aetiology of illicit drug initiation and subsequent misuse in women. Br J Psychiatry.

[B41] Agrawal A, Lynskey MT (2006). The genetic epidemiology of cannabis use, abuse and dependence. Addiction.

[B42] Fidler JA, West R, Jaarsveld CHM, Jarvis MJ, Wardle J (2008). Smoking status of step-parents as a risk factor for smoking in adolescence. Addiction.

[B43] Hemmingsson T, Lundberg I (2005). How far are socioeconomic differences in coronary heart disease hospitalization, all-cause mortality and cardiovascular mortality among adult Swedish males attributable to negative childhood circumstances and behaviour in adolescence?. Int J Epidemiol.

